# Functionalized organosolv lignin grafted with 3-aminopropyltriethoxysilane: A bio-based adsorbent for phosphate recovery from dairy wastewater

**DOI:** 10.1016/j.heliyon.2025.e42559

**Published:** 2025-02-08

**Authors:** Minu Masliha, Mukesh Padnekar, Jessica De Micco, Siva Ponnupandian, Kona Mondal, Ramesh Babu Padamati

**Affiliations:** a School of Chemistry, CRANN, Trinity College Dublin, D02 PN40, Dublin, Ireland; b AMBER, SFI Research Centre for Advanced Materials and BioEngineering Research, Ireland SFI Research Centre, Trinity College Dublin, D02 PN40, Dublin, Ireland; c School of Physics, CRANN, Trinity College Dublin, D02 PN40, Dublin, Ireland; d Dairy Processing Technology Centre (DPTC), University of Limerick, V94 T9PX, Limerick, Ireland

**Keywords:** Organosolv lignin, 3-Aminopropyltriethoxysilane, Adsorption, Chemisorption

## Abstract

Wastewater rich in phosphates and nitrates causes eutrophication and leads to the impairment of freshwater resources. Out of various methods used, adsorption is the immaculate and economical for removing and recovering phosphates and nitrates from wastewater streams in a single-step process. This study explores the potential of bio-based adsorbent, functionalized organosolv lignin [OL], chemically modified by grafting with 3-aminopropyltriethoxysilane [APTES], as an effective bio-based adsorbent [OL-APTES-H+] for phosphate recovery from aqueous solutions and industrial wastewater. The characterization of OL-APTES-H+ was performed using multiple analytical techniques, providing comprehensive information on the material morphology, elemental composition, functional groups, thermal stability, surface charge, and electrokinetic behavior. The adsorption efficiency of OL-APTES-H+ was assessed under varying experimental conditions, including pH, contact time, and initial phosphate concentration. The adsorption capacity of OL-APTES-H+ depended on pH, with different forms of phosphate species being preferentially adsorbed at different pH values. A maximum adsorption capacity of 21.12 mg/g was achieved at pH 5. Kinetic studies indicated that the adsorption process followed a combination of electrostatic interactions, chemisorption and surface interaction, as evidenced by SEM and EDS analyses. XPS results confirm phosphorus incorporation on the adsorbent surface, reinforcing chemisorption. Adsorption isotherm analysis revealed that the data fitted well to the Langmuir isotherm model, suggesting a monolayer adsorption mechanism. The adsorption performance of OL-APTES-H+ was enhanced in the presence of monovalent ions, while a slight reduction in efficiency was observed in the presence of divalent anions. When applied to industrial dairy wastewater, OL-APTES-H+ exhibited phosphate removal efficiencies ranging from 30 % to 58 %. Overall, OL-APTES-H+ demonstrates considerable potential as a bio-based adsorbent for phosphate recovery, effectively mitigating environmental pollution in wastewater bodies and providing an eco-friendly source of phosphates for sustainable agricultural practices.

## Highlights

• First time demonstration of functionalized organosolv lignin for phosphate adsorption and recovery from industrial dairy wastewater.
• Sustainable alternative to conventional inorganic and metal-based adsorbents, reducing environmental impact and toxicity.
• High phosphate removal efficiency confirmed in real time dairy wastewater, supporting its potential for wastewater treatment.

## Graphical abstract

**Figure:**
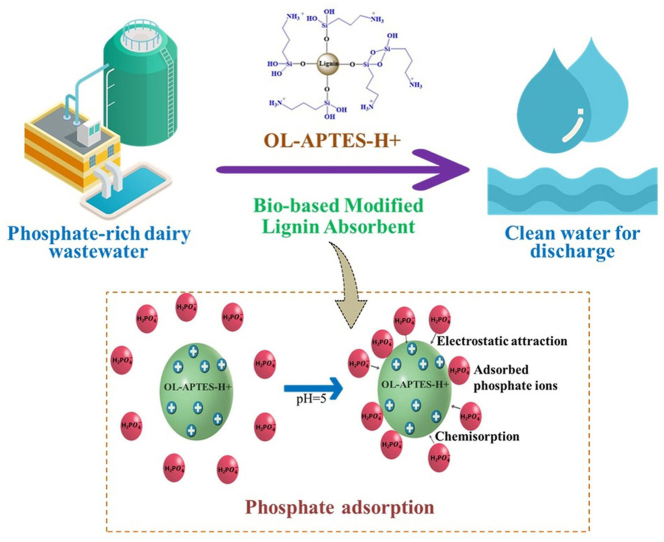

## 1 Introduction

Water contamination and diminishing drinking water resources have heightened interest in wastewater treatment to improve water availability and quality. In particular, dairy industries generate massive wastewater discharge streams from cleaning, changing product type, and milk processing. In dairy processing plants, water quality degradation is a significant concern, with 50–80 % of the total water usage being adversely affected. This means that only 20–50 % of the water remains clean and suitable for reuse without extensive treatment [1]. The volume of wastewater generated in dairy processing plants, which varies from 0.2 to 10 L per litre of processed milk, is influenced by factors including factory size, good manufacturing practices, clean-in-place procedures, and technological advancements [1,2]. Dairy process effluent streams are rich in carbon, nitrogen, phosphorus, and other minerals, with phosphorus being a key concern. Removing phosphates from wastewater is critical for both environmental protection and resource conservation. Although essential for plant growth and food production, phosphates pose a significant threat to aquatic ecosystems when present in excess, contributing to eutrophication, harmful algal blooms, and oxygen depletion in water bodies [2]. Additionally, as a non-renewable resource, phosphorus is rapidly depleted due to its extensive use in industrial agriculture to support the growing global population [3]. Therefore, efficient phosphorus removal and recovery from wastewater is vital to prevent environmental pollution and to conserve this valuable resource for future use.

Various methods are employed to treat dairy wastewater and facilitate its reuse by mitigating water toxicity. These methods encompass chemical precipitation [4], reverse osmosis [5], and biological removal [6]. Chemical precipitation is widely adopted due to its high efficiency and straightforward operation; however, the need for large amounts of chemicals often leads to contaminated sludge, which is a challenge to manage. Moreover, this method is highly dependent on pH levels [7]. Membrane-based reverse osmosis is effective in certain scenarios, however, it is restricting widespread adaptation due to high costs and limited selectivity [8]. Despite their low operational costs and minimal byproduct generation, biological methods are less favourable due to their operational complexities, extended retention times, and reduced effectiveness at low phosphate concentrations [9]. On the other hand, adsorption is a promising method for phosphate removal due to its advantage of not generating sludge, cost-effectiveness, high selectivity, low energy requirements, and operational simplicity [[10], [11], [12]]. Common phosphate removal adsorbents, such as biomass, clay minerals, metal oxides and ion-exchange resins often suffer from several limitations, typically exhibit low adsorption capacities, and incur high doping costs. Additionally, they pose risks of secondary pollution and present significant challenges in terms of recycling. These drawbacks underscore the need to develop low-cost, environmentally friendly adsorbents with enhanced phosphate adsorption capabilities [13,14]. Current industrial P removal methods, which use metal-based compounds and lime [Ca(OH)_2_] to precipitate P as calcium phosphate, generate significant amounts of metal-laden sludge, highlighting the need for efficient disposal solutions [15].

Lignin, the second most abundant renewable biopolymer, can readily be extracted from woody biomass and paper processing waste side streams [16]. Although studies have been conducted on sustainable upgrading, its reactive groups make it a viable source for low-carbon footprint products, and the production of adsorbents from lignin has received much attention in recent years [17]. Despite extensive recent research on lignin efficacy as an adsorbent for heavy metals and colour dyes [[18], [19], [20]], there remains a significant gap in studying the potential of lignin for phosphate removal from wastewater. Lignin exhibits a low adsorption capacity towards phosphates [1.92 mg P g⁻^1^] due to the presence of a negatively charged functional group [-OH], which creates electrostatic repulsion against the negatively charged phosphate ions [21]. Therefore, modifying lignin is essential to enhance its phosphate adsorption capacity. Metal oxide modification is widely recognized as an effective method to enhance the phosphate adsorption capacity, with common modifications including the incorporation of La(III) oxide, Zr(IV) oxide, and Fe(III) oxide [[22], [23], [24]]. However, these adsorbents have the potential drawbacks of higher adsorption costs and the leaching of metal ions, which could contribute to secondary water pollution. Wang et al. [25] used industrial alkali lignin treated at high temperatures, followed by amination with formaldehyde and N,N-dimethylformamide. The quaternary amination of the lignin was achieved with epichlorohydrin to produce a metal-free lignin-based adsorbent. Despite its effectiveness, this process involved hazardous chemicals and complex steps. Thus, there's a need for simpler lignin modification methods using safer and greener chemicals to enhance sustainability and application potential of the natural based adsorbents.

This study presents the development of an organosolv lignin-based adsorbent functionalized with APTES for phosphate removal, an approach utilized for the first time. APTES was chosen to modify OL as it introduces amine [-NH₂] groups that enhance phosphate adsorption. Upon protonation, these groups form positively charged [-NH₃⁺] sites, improving electrostatic interactions. Additionally, APTES facilitates silane grafting, enhancing the stability and reusability of the modified lignin. This method eliminates the need for pretreatment steps, resulting in reduced energy consumption, minimized use of hazardous chemicals, and a cost-effective process. The study's main objectives include: (1) Functionalizing the OL through chemical grafting using APTES. (2) Characterizing the modified lignin through Fourier transform infrared spectroscopy [FTIR], thermogravimetric analysis [TGA], scanning electron microscopy [SEM], energy-dispersive X-ray spectroscopy [EDS], X-ray photoelectron spectroscopy (XPS), zeta potential analysis, and point of zero charge measurements. (3) Investigating the effects of adsorbent dosage, pH, and initial phosphate concentration. (4) Understanding the adsorption mechanism through reaction kinetics studies and isotherm models. (5) Understanding the adsorption behavior in the presence of coexisting ions and conducting desorption studies. (6) Evaluating the phosphate removal efficiency of the developed bio-based adsorbent using industrial dairy wastewater samples, demonstrating its potential as a practical and environmentally sustainable solution for wastewater treatment.

## 2 Materials and methods

### 2.1 Materials

The OL was supplied by Chemical Point UG [Deisenhofer, Germany] and used without any pre-treatment. Aminopropyltriethoxysilane [APTES, 98 %], absolute ethanol [EtOH, 99 %], and hydrochloric acid [37 %] were purchased from Merck [Ireland] and used as supplied. Potassium dihydrogen phosphate [KH_2_PO_4_] was purchased from Fisher Chemical [UK], and all solutions were prepared in distilled water.

### 2.2 Amine silanization functionalization of lignin

**Scheme 1:**
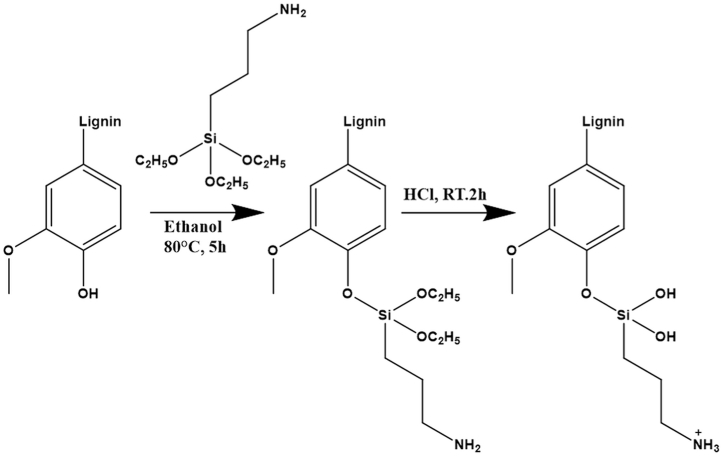
Chemical grafting of APTES on organosolv lignin and protonation.

The OL was chemically modified via grafting using APTES. Firstly, 5 g of the OL and 5 g of the APTES were placed into a 250 mL round bottom flask with 100 mL of ethanol. The reaction mixture was stirred at 80 °C for 5 h using an oil bath [26]. The produced dark brown APTES grafted lignin [OL-APTES] was filtered under vacuum and washed multiple times with ethanol and water to remove the unreacted APTES at room temperature, followed by drying at 60 °C overnight. Then, the dried OL-APTES [5 g] was treated with 0.5 mol/L HCl for 2 h to protonate the amine groups [-NH₂] into positively charged ammonium [-NH₃⁺] groups grafted onto the APTES-modified lignin [27]. The obtained adsorbent [OL-APTES-H+ ] was dried at 60 °C [Scheme 1].

### 2.3 Characterisation of the materials

The chemical structure of the modified lignin samples was analyzed by FTIR spectroscopy [PerkinElmer, Diamond/KRS-5 sandwich assembly, USA] in the attenuated total reflectance mode. The spectra were recorded from 4000 to 600 cm^−1^, with 16 accumulations and a resolution of 4 cm^−1^. SEM micrographs were captured using a Zeiss Ultra Plus SEM. XPS measurements were performed [Kratos AXIS ULTRA DLD instrument] with a monochromatic Al Kα source [1486.58 eV, 300 W] to measure the contents of different elements [C, H, N and P]. Survey spectra were recorded at pass energy of 160 eV [1 eV step, 50 ms dwell], and high-resolution scans at 20 eV [0.05 eV step, 100 ms dwell]. Samples were dusted and compacted onto double-sided adhesive tape. The C 1s peak at 284.8 eV was used for charge referencing, and the detection limit was ∼0.1 at%. Spectra were processed using a Shirley background and mixed Gaussian-Lorentzian peaks, with relative sensitivity factors from the CasaXPS library. Thermogravimetric analysis [PerkinElmer Pyris 1 TGA, USA] was used to measure the thermal properties of the modified lignin products. Each sample [∼3 mg] was put into a platinum pan and heated at 10 °C/min under a nitrogen environment from 30 to 800 °C. The zeta potential of the compounds was measured using zetasizer [Malvern Zetasizer Nano-ZS] to determine the charge on various modified lignins. To measure the zeta potential, the functionalized lignin samples were dispersed in solutions with varying pH levels and were pH adjusted using 0.05 M HCl and NaOH solutions. The pH values at the point of the zero charge [pH_pzc_] for OL, OL-APTES, and OL-APTES H+ were measured by dispersing 0.1 g in different pH [[2], [3], [4], [5], [6], [7], [8], [9], [10], [11], [12]] solutions of 10 ml 0.01 M NaCl solutions. The pH was adjusted using 0.05 M HCl and NaOH solutions. The mixtures were magnetically stirred for 48 h. After 48 h, the mixtures were filtered, and the filtrate pH was measured. The difference between the initial pH and final pH [ΔpH = pH_i_-pH_f_] is plotted against the initial pH. The pH_pzc_ was measured at the intersection of the resulting curve where ΔpH = 0 from the graph [28]. The phosphate analysis [Hach Spectrophotometer, S-FIEP1-DR3900, UK] was carried out using Hach LCK349 Phosphate [Ortho/Total] cuvette test kits [0.05–1.5 mg/L PO₄-P] and LCK350 Phosphate [ortho/total] cuvette test kits [2.0–20.0 mg/L PO₄-P].

### 2.4 Adsorption experiments and kinetics, equilibrium, and thermodynamics

Various factors, including adsorbent dosage, pH and phosphate concentration were investigated in the adsorption experiments using OL-APTES-H+. Modified lignin OL-APTES-H+ was added to 10 mL of phosphate solution contained within a centrifuge tube for all the adsorption studies. The mixture was thoroughly agitated and subsequently subjected to continuous stirring on a roller shaker operating at a speed of 70 rpm. After the stipulated time, the mixtures were filtered using a 0.45 μM nylon syringe filter and the phosphate concentration in the filtrate was determined using a Hach spectrophotometer with LCK349 and LCK350 cuvette test kits, which are suitable to measure medium to high range [1.5 mg/L- 20.0 mg/L] phosphate concentrations. The effect of different adsorbent dosages was studied by varying from 0.5 g/L to 6 g/L in 10 mL of the 50 mg/L of the phosphate solution for 1 h at room temperature. A time-based adsorption analysis was conducted over a time duration ranging from 10 min to 24 h. A series of phosphate solutions with concentrations ranging from 5 mg/L to 400 mg/L were prepared. The phosphate adsorption capacity of OL-APTES-H+ was then analyzed across these varying initial phosphate concentrations. The pH dependency of the adsorption capacity was investigated by adjusting the pH of a 50 mg/L phosphate solution over a range from pH 2 to pH 12 by adjusting the pH using 0.05 M HCl and 0.05 M NaOH solutions. The pH-adjusted phosphate solutions were mixed with OL-APTES-H+ at a dosage of 0.5 g/L, and the mixtures were stirred for 1 h at room temperature.

The adsorption capacity [q_e_] and the percentage removal rate [R] of all the adsorption experiments were calculated using Eqs. (1), (2) respectively.

(1) $$q_{e}=\frac{C_{0}-C}{M}\times V$$

(2) $$R\left(\%\right)=\frac{C_{0}-C}{C_{0}}\times 100$$

Here C_0_ and C are the initial and final concentrations of phosphate solutions in the adsorption experiments. M is the OL-APTES-H+ mass in g, and V is the phosphate solution volume in L.

Adsorption kinetics studies were conducted using two different phosphate solution concentrations [30 mg/L and 50 mg/L] at pH 5. The experiments were performed with an adsorbent dosage of 0.5 g/L at room temperature. Aliquots were collected at 10-min intervals for the first hour and at 30-min intervals for the subsequent 3 h, resulting in a total experiment duration of 4 h. The adsorption capacity values were fitted to the adsorption kinetics models [Eqs. (3), (4)] and the intraparticle diffusion model [Eq. (5)].

(3) $$q_{t}=q_{e}\left(1-\exp\left(-k_{1}t\right)\right)$$

(4) $$q_{t}=\frac{{K_{2}q}_{e}^{2}t}{1+K_{2}q_{e}t}$$

Here q_t_ and q_e_ are the adsorption capacities at time t and equilibrium [mg/g] respectively. The symbols k_1_ and K_2_ [min ^−1^] are the pseudo-first-order rate constant and pseudo-second-order equilibrium rate constant.

(5) $$q_{t}=K_{id}t^{1/2}+C_{i}$$

The K_id_ [mg/g.min^1/2^] is the intra-particle diffusion rate constant and C_i_ [mg/g] represents the effect of the boundary layer on the adsorption rate.

The adsorption isotherm model [Eqs. (6), (7)] was fitted to the data obtained by conducting adsorption trials in different initial concentrations of phosphate solution at three different temperatures [25 °C,35 °C,45 °C] and sampling after 4.5 h.

(6) $$q_{e}=\frac{q_{\max}K_{L}C_{e}}{1+K_{L}C_{e}}$$

(7) $$q_{e}=K_{F}C_{e}^{1/n}$$

Here q_max_ is the saturation adsorption capacity [mg/g], K_L_ and K_F_ denote Langmuir and Freundlich isotherm model constants, C_e_ denotes the equilibrium adsorption capacity [mg/L], q_e_ [mg/g] denotes the adsorption capacity at time t, and n denotes the adsorption strength parameter.

To analyse the adsorption mechanism, the changes in free energy, heat, and entropy were estimated using thermodynamic equations [Eqs. (8), (9), (10), (11)]:

(8) $$K_{a}=\frac{1000\;K_{L}MC}{\gamma }$$

(9) $$\Delta G{}^{\circ}=-RTlnK_{a}$$

(10) ΔG°=ΔH°−TΔS

(11) $$\ln\;K_{a}=\frac{\Delta S{}^{\circ}}{R}-\frac{\Delta H{}^{\circ}}{RT}$$

Here M [g/mol] is the adsorbate's molar mass, C is the adsorbate's standard concentration [1 mol/L], γ is the activity coefficient [dimensionless], and K_a_ is the thermodynamic non-dimensionless standard equilibrium constant. T [K] is the temperature, and R [8.314 J/mol. K] is the universal gas constant.

### 2.5 Desorption experiments

Desorption studies were performed to evaluate the reusability of OL-APTES-H+ adsorbent using 0.1 mol/L sodium hydroxide as the desorbing agent. Phosphate-saturated OL-APTES-H+ was applied at a dosage of 0.5 g/L and immersed in the NaOH solution, with desorption experiments conducted at intervals of 1, 3, and 4 h. The mixtures were stirred at 70 rpm using a roller shaker to ensure uniform desorption. At each time point, samples were collected, filtered, and analyzed for phosphate concentration using Hach LCK349 Phosphate [Ortho/Total] cuvette test kits. Desorption efficiency was calculated using Eq (12), demonstrating the potential of OL-APTES-H+ for sustainable reuse in phosphate recovery and nutrient recycling applications.

(12) $$Percent\;of\;desorption=\frac{Amount\;of\;desorbed\;phosphate\;}{Amount\;of\;absorbed\;phosphate\;}\times 100$$

## 3 Results and discussion

### 3.1 Material characterization before and after functionalization

**Fig. 1:**
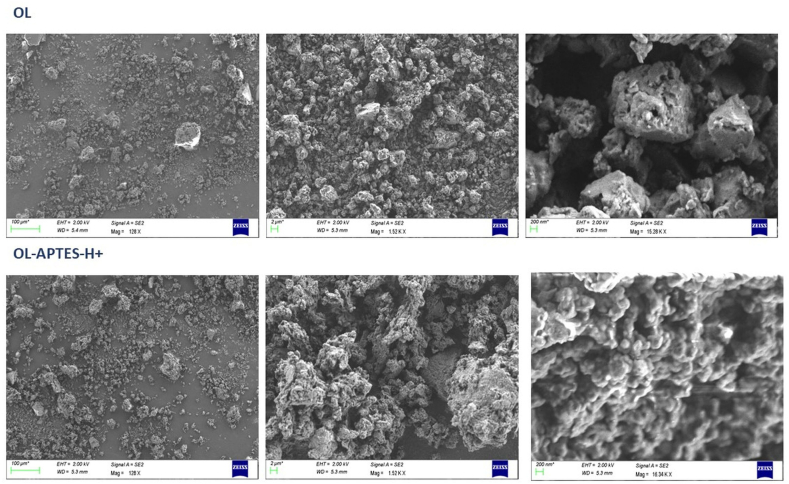
SEM images of OL and OL-APTES-H+.

The structural and chemical properties of both OL and OL-APTES-H+ were systematically characterized to evaluate the impact of functionalization on surface morphology, elemental composition and surface charge. The surface morphology of OL and OL-APTES-H+ was analyzed through SEM microscopy, revealing significant variations in their structural features, as depicted in Fig. 1. The surface of OL exhibited a notably rough and irregular texture, characterized by an uneven distribution of features, indicating a heterogeneous structure. In contrast, the OL-APTES-H+ sample, which had undergone silane grafting, displayed the presence of distinct foils on its surface, indicating successful chemical modification. Additionally, post-treatment with HCl led to the formation of visible holes in the APTES foils, a phenomenon that can be attributed to the strong and localized interaction between the amino groups present in APTES and the acidic protons introduced by the HCl. This interaction is believed to play a key role in altering the surface structure and morphology of the material [29].

**Fig. 2:**
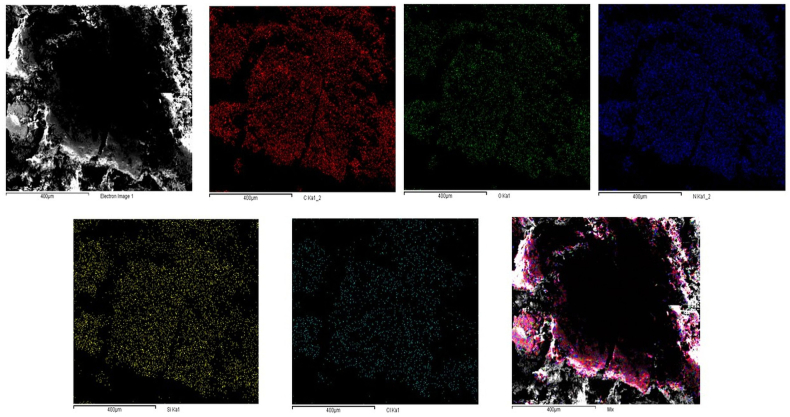
SEM-EDS mapping of OL-APTES-H+.

An EDS analysis was performed to further confirm the successful APTES grafting onto the OL surface. The elemental mapping obtained from the EDS analysis demonstrated the presence of silicon, nitrogen, and chlorine atoms on the OL-APTES-H+ surface, providing strong evidence of the APTES grafting and subsequent HCl treatment. The detection of silicon and nitrogen atoms corroborates the incorporation of the APTES moiety, while the presence of chlorine signals the protonation of the amino groups due to the HCl treatment [Fig. 2].

**Fig. 3:**
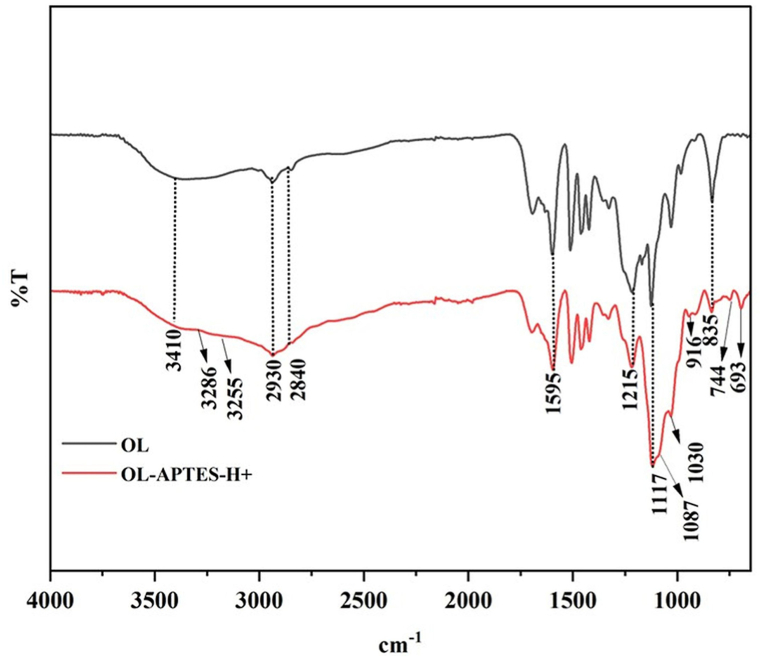
FTIR spectrum of OL and OL-APTES-H+.

Fig. 3 illustrates the FTIR spectral analysis, which reveals various lignin characteristic peaks in both OL and OL-APTES-H+ samples. These include the broad -OH stretching vibration at 3410 cm⁻^1^, and the C-H asymmetric and symmetric stretching vibrations corresponding to -CH₃ and -CH₂- groups at 2930 cm⁻^1^ and 2840 cm⁻^1^, respectively. The absorption band at 1595 cm⁻^1^ is attributed to the aromatic ring, while the peak at 1215 cm⁻^1^ corresponds to the aromatic nucleus C-O vibrational mode. A unique peak at 1117 cm⁻^1^ is associated with ring breathing vibrations involving C-C, C-O, and C=O stretching. Furthermore, the peak at 835 cm⁻^1^, observed in both samples, indicates C-H out-of-plane deformation within the aromatic ring structure. These characteristic lignin absorption peaks confirm that the lignin modification procedure preserved the core aromatic structure of OL, without disrupting its skeletal framework [30].

In the OL-APTES-H+ samples, the peaks at 3286 cm⁻^1^ and 3255 cm⁻^1^ correspond to the asymmetric and symmetric stretching modes of NH₂ groups, respectively [31]. New peaks observed at 1030 cm⁻^1^ and 916 cm⁻^1^ are attributed to the formation of Si-O-Si and Si-O-C bonds, further supporting the successful chemical modification of OL [32]. The presence of bands at 744 cm⁻^1^ and 693 cm⁻^1^, assigned to the stretching vibrations of Si-C bonds, further confirms the introduction of propylsilane functionalities. The peak observed at 1087 cm⁻^1^, corresponding to the stretching vibrations of the C-N bond, further validates the presence of the amine groups from APTES grafting. Additionally, a noticeable reduction in the intensity of the -OH peak, attributed to the reaction between the hydroxyl groups of lignin and APTES during the silanization process, was observed [33]. These characteristic peaks strongly indicate the successful grafting of APTES onto the OL surface.

**Fig. 4:**
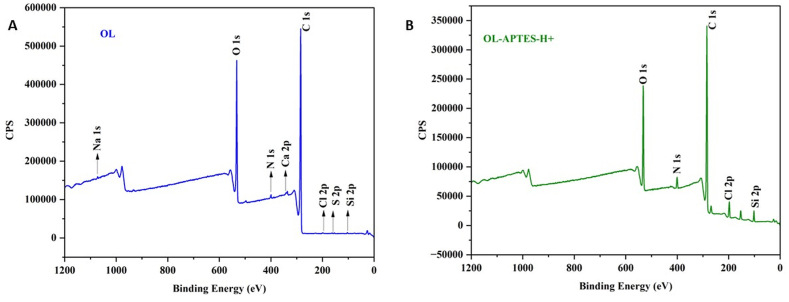
XPS spectra of OL (A) and OL-APTES-H+ (B).

Fig. 4 presents the XPS analysis of OL and OL-APTES-H+, revealing the surface chemical composition. The deconvoluted high-resolution C 1s spectrum of OL revealed four chemical environments: C=C/C–C [284.8 eV], C–OH/C–O–C/C–N [286.0 eV], O–C–O/C=O [287.0 eV], and O–C=O [288.7 eV], characteristic of lignin. The O 1s spectrum displayed peaks at 533.4 eV, 535.3 eV, and 537.3 eV, attributed to Ph–O/Ph–C–O/O–O, C=O/O–C–OH, and Ph–OH/C–O bonds, respectively [34,35]. Trace amounts of nitrogen [0.77 %], sodium [0.11 %], sulfur [0.13 %], silicon [0.37 %], chlorine [0.15 %], and calcium [0.57 %] were also detected, consistent with the untreated nature of OL.

In contrast, the survey spectrum of OL-APTES-H+ confirmed successful functionalization. A Si 2p peak at 102 eV, along with C 1s [284.7 eV], O 1s [532.8 eV], and N 1s [401.5 eV] signals, was observed. Quantitative analysis showed a decrease in carbon content from 76.46 % [OL] to 73.22 % [OL-APTES-H+], with an increase in nitrogen content to 2.91 % and silicon at 4.01 %. A Cl 2p peak at 199.2 eV [2.70 %] confirmed the presence of chloride ions. The deconvoluted N 1s spectrum exhibited peaks at 399.6 eV and 401.5 eV, corresponding to C–N and protonated N⁺ species, respectively. These results confirm the successful functionalization of OL with APTES and subsequent protonation with HCl. The high-resolution spectra are given in the supporting information [Fig. S1].

The TG and DTG curves of OL, both before and after APTES modification, were analyzed to evaluate the changes in thermal stability following the grafting process [Fig. S2]. The weight loss curve of OL showed a distinct deviation from that of OL-APTES, indicating altered thermal degradation behavior. Both samples exhibited a three-stage decomposition process. The TGA analysis revealed differences in thermal behavior between OL and OL-APTES, particularly concerning water evaporation and subsequent thermal degradation. An initial weight loss in both samples was observed below 100 °C, attributed to moisture evaporation. For OL, a substantial weight loss of 48 % occurred within the temperature range of 200 °C–600 °C, with a peak maximum weight loss at 337.4 °C. This degradation is associated with the breakdown of the lignin matrix and the volatilization of phenolic compounds. Due to the complexity of lignin's macromolecular structure, the degradation process extended over a broad temperature range [36]. In contrast, OL-APTES exhibited a lower weight loss of 36 % over the same temperature range, with a maximum weight loss occurring at a higher temperature of 401 °C. Furthermore, the residue content at 800 °C for OL and OL-APTES was 36 % and 47 %, respectively, demonstrating that the OL-APTES had enhanced thermal stability [37]. These findings suggest that APTES grafting significantly improves the thermal stability of lignin, making it a more suitable candidate for applications requiring materials with increased thermal resistance.

**Fig. 5:**
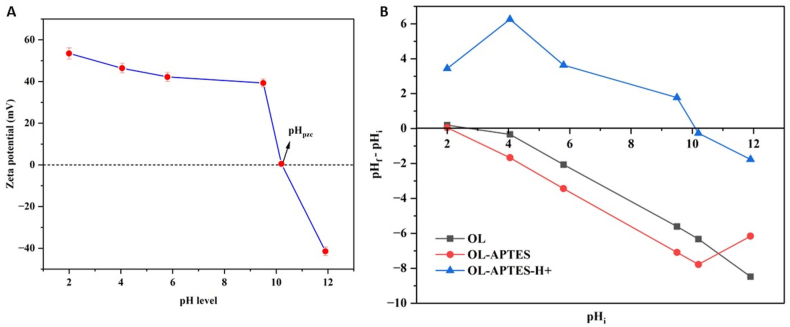
Zeta potential measurements of OL-APTES-H+ (A), and point of zero charge density profiles of OL, OL-APTES, and OL-APTES-H+(B).

Zeta potential studies of OL-APTES-H+ were performed across a range of pH values to assess the variation in surface charge density [Fig. 5]. This analysis is critical for understanding the potential adaptability of modified lignin in wastewater treatment, as different wastewater streams exhibit varying pH conditions. The zeta potential results indicated a strongly positive surface charge, with values ranging from +54 mV to +40 mV across a broad pH range of 2–9.5, demonstrating notable stability and consistent surface charge retention under varying pH conditions. However, at pH levels exceeding 10, there was a sharp decline in the zeta potential, indicating a loss of surface charge density under highly basic conditions. Additionally, a comparative zeta potential analysis of the dispersions of OL, OL-APTES, and OL-APTES-H+ in distilled water yielded values of −30 mV, −51.7 mV, and +45.3 mV, respectively. These results confirm the successful amine silanization of OL to form OL-APTES, and the subsequent formation of positively charged NH₃⁺ groups on OL-APTES-H+ upon protonation. The strong positive charge exhibited by OL-APTES-H+ over a wide pH range indicates its potential for efficient adsorption in diverse wastewater environments, where pH plays a crucial role in influencing adsorption efficiency. This persistent positive charge over a broad pH range also reduces the need for frequent pH adjustments, thereby lowering operational costs and enhancing the scalability of adsorption processes [26].

The pH_PZC_ was also determined, representing the pH at which the net surface charge of the adsorbent is zero. At pH values higher than the pH_PZC_ the adsorbent surface carries a negative charge, while at pH values lower than the pH_PZC_, it becomes positively charged [38]. The analysis revealed that the pH_PZC_ for OL and OL-APTES is approximately 2.1, whereas for OL-APTES-H+, it shifted to pH 10 [Fig. 5]. This significant shift in the pH_PZC_ is consistent with the zeta potential data, further confirming the protonation of the amine groups and the resulting positive surface charge over a broad pH range.

In summary, the data from SEM, EDS, FTIR, TG-DTG, and zeta potential analyses confirm the successful functionalization of OL with APTES, resulting in a stable, positively charged material. The modified lignin retains its structural integrity and exhibits enhanced thermal stability and surface charge across a wide pH range. These characteristics make it a promising adsorbent for wastewater remediation, offering improved efficiency, scalability, and environmental sustainability for industrial applications.

### 3.2 Adsorption study

#### 3.2.1 Parameter effects

**Fig. 6:**
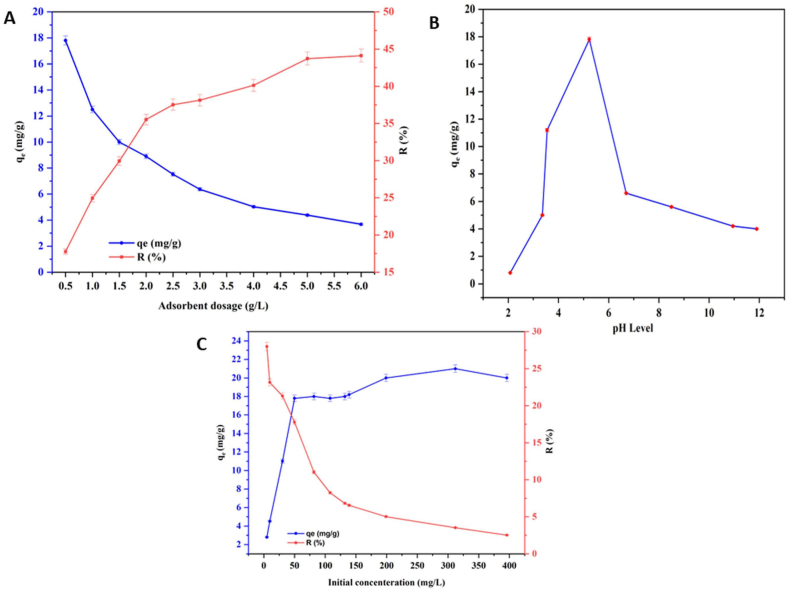
Effect of adsorption parameters: (A)Adsorbent dosage (50 mg/L phosphate concentration, pH = 5), (B) pH (0.5 g/L adsorbent dosage in 50 mg/L phosphate concentration), (C) initial concentration of phosphorous (pH = 5).

Fig. 6 illustrates the impact of varying dosages of OL-APTES-H+ on the adsorption capacity and the removal efficiency of phosphate ions from an aqueous solution. OL-APTES-H+ dosages ranging from 0.5 g/L to 6 g/L are added to a 50 mg/L phosphate solution, and the adsorption capacity and removal rate values are measured after 1 h. It is observed that the q_e_ value gradually decreases while the removal rate increases. The optimal adsorption capacity was determined to be 17.8 mg/g at a dosage of 0.5 g/L, while the highest phosphate removal efficiency, reaching 44 %, was achieved at a dosage of 6 g/L. The pH plays an integral role in the phosphorus morphology and surface charge density of OL-APTES-H+. As mentioned, the OL-APTES-H+ showed a positive charge over a pH range [2–9.5], affecting the electrostatic attraction or electrostatic repulsion of P in the solution [39]. The adsorption studies were conducted over a pH range of 2–12, and the maximum adsorption capacity was observed around pH 5 [see Fig. 6]. Phosphate exists in solution in different forms, including H_3_PO_4_, H_2_P $O_{4}^{-}$, H_2_P $O_{4}^{2-}$ and P $O_{4}^{3-}$. The distribution of these species is governed by dissociation constants: H_3_PO_4_ dissociates into H_2_P $O_{4}^{-}$ with a dissociation constant pK1 = 2.12, H_2_P $O_{4}^{-}$ further dissociates into H_2_P $O_{4}^{2-}$ at pK2 = 7.21, and H_2_P $O_{4}^{2-}$ dissociates into P $O_{4}^{3-}$ at pK3 = 12.67. These constants regulate the proportions of each species in solution depending on the pH [40]. Due to the different forms of phosphorus in the solution at different pH levels, the q_e_ decreased drastically at highly acidic pH [pH < 2] and at pH values above 7 [41]. In acidic conditions, H_3_PO_4_ is the dominant species, exhibiting weak interaction with the adsorbent sites. As the solution pH increases, H_2_P $O_{4}^{-}$ becomes the predominant phosphate compound, alongside a smaller fraction of undissociated H_3_PO_4_. At these pH levels, the OL-APTES-H+ binding sites are protonated, enhancing electrostatic attraction between the positively charged adsorbent and the negatively charged H_2_P $O_{4}^{-}$ ions. This increased coulombic interaction and chemisorption facilitate higher phosphate uptake [42]. However, as the pH increases further, the electrostatic component of the adsorption force diminishes, leading to a gradual reduction in phosphate adsorption. In alkaline conditions, the reduced adsorption capacity is also attributed to the competitive adsorption between phosphate anions and hydroxide ions, which occupy the available binding sites [43]. Since the pH_pzc_ of OL-APTES-H+ is at 10, the adsorbent will have a negative charge at a pH higher than 10 in the phosphate solution, thereby hampering the adsorption capacity and resulting in a low q_e_ value. The phosphate in the studied system at pH 5 was present primarily as dihydrogen phosphate ions. The positive zeta potential of OL-APTES-H+ at pH 5 corresponded with the highest observed adsorption efficiency for the H_2_P $O_{4}^{-}$ ions. The pH dependence of the zeta potential suggests that the electrostatic interactions between the OL-APTES-H+ and H_2_P $O_{4}^{-}$ are optimized at this pH level, leading to maximum adsorption capacity [44]. The effect of the initial concentration of P solution on the adsorption capacity was studied. This variation in P concentrations affects the potential energy of the solvent, consequently influencing the adsorption capacity. The removal rate of P continuously dropped as the concentration increased from low to high. When attempting adsorption at a low P concentration [5 mg/L], the removal rate was 28 %, demonstrating the acceptable capacity of this biobased adsorbent for phosphate removal. The maximum adsorption capacity was observed at a P concentration of 312 mg/L [q_e_ = 21.12 mg/g], slightly higher than the qe value observed for 50 mg/L [17.8 mg/g]. This suggests that up to a P solution concentration of 50 mg/L, the OL-APTES-H+ adsorption efficiency increased considerably, and subsequent increases in P concentrations resulted in minimal changes in the q_e_ value.

#### 3.2.2 Adsorption model

**Fig. 7:**
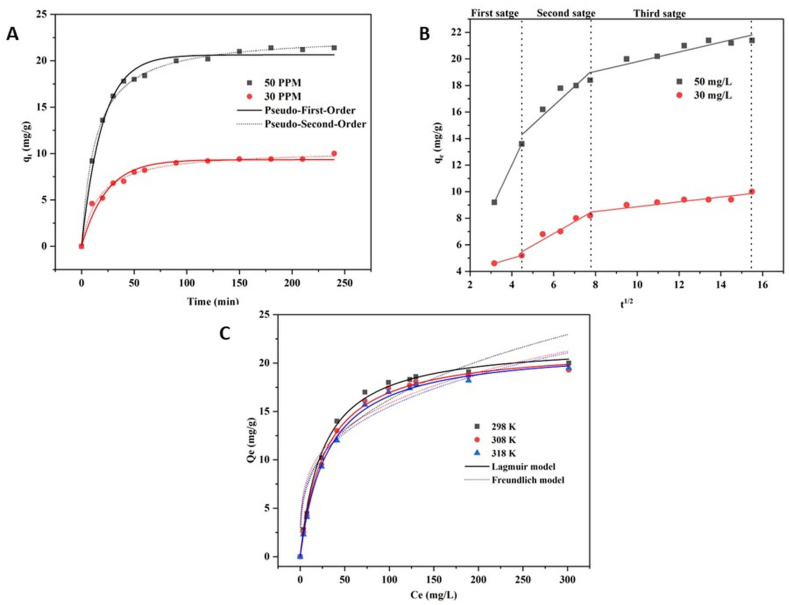
Kinetic model (A), Intraparticle diffusion model (B), and isotherm model (C).

The obtained data were fitted to the pseudo-first-order and pseudo-second-order equations to investigate the reaction kinetics of the adsorption mechanism. The plot and data of reaction kinetics are shown in Fig. 7 and Table 1. OL-APTES-H+ reached adsorption equilibrium after 60 min from the start of the experiment. As these data over 240 min fit into the kinetics model, the correlation coefficient [R^2^] value is higher and closer to 1 in the pseudo-second-order kinetic model compared to the pseudo-first-order kinetic model. As a result, it proved that chemisorption dominates the adsorption mechanism, which is complemented by physical adsorption [45].

**Table 1:** Kinetic model parameters of P adsorption by OL-APTES-H+.

P concentration (mg/L)	q_exp_ (mg/g)	Pseudo first order			Pseudo second order		
		k_1_	q_1e_(mg/g)	$R_{1}^{2}$	k_2_	q_2e_ (mg/g)	$R_{2}^{2}$
30	9.41	0.042	9.336	0.965	0.006	10.371	0.987
50	20.23	0.050	20.640	0.986	0.003	22.780	0.996

The supporting information [Table S1] compares the kinetic data with reported adsorbents for phosphate adsorption from the literature. All results were consistent with the pseudo-second-order reaction model, which aligns with this study's findings.

The intraparticle diffusion model was used to better understand the phosphate removal adsorption mechanism [46]. The data obtained are shown in Fig. 7 and Table 2. This indicates that the adsorption process is multi-step and depends on several factors. The initial 20 min of the adsorption is considered the first stage, where the surface charge density plays a major role and facilitates the electrostatic adsorption and chemisorption process. In this rapid adsorption stage, the potential energy difference is high between H_2_P $O_{4}^{-}$ and OL-APTES-H+ in the solution, which causes the rapid movement of anion to the cationic adsorbent and shows a higher slope in the adsorption curve with surface diffusion as a dominant effect [47]. During the second stage [20–60 min] the curve is flatter, indicating slower adsorption. The difference between the potential energy and phosphate concentration in the solution decreases during this stage, the adsorption sites on OL-APTES-H+ are mostly occupied, and intraparticle diffusion mostly limits adsorption compared to the quick initial stage [48]. After 60 min, the adsorption has reached a dynamic equilibrium, with equal adsorption and desorption rates of phosphates. Thus, the adsorption process is stabilised at this point.

**Table 2:** Intraparticle diffusion model of P adsorption by OL-APTES-H+.

P (mg/L)	K_id, 1_ (mg/g.min^1/2^)	R^2^	K_id, 2_ (mg/g.min^1/2^)	R^2^	K_id, 3_ (mg/g.min^1/2^)	R^2^
50	3.359	1	1.440	0.849	0.365	0.835
30	0.458	1	0.896	0.916	0.182	0.823

Adsorption was also performed at pH = 6 with a dosage of 0.5 g/L in different concentrations of the P solution at three different temperatures [Fig. 7 and Table 3]. The adsorption capacity is lower at higher temperatures, while lower temperatures boost adsorption efficiency. The data obtained were fitted to the Langmuir and Freundlich isotherm models. The correlation coefficient $R_{L}^{2}$ is 0.99, which is greater than the $R_{F}^{2}$ value of 0.87–0.92. This indicated that the data fitted better with the Langmuir model and thus showed a regular single molecule layer adsorption process of phosphates on OL-APTES-H+.

**Table 3:** Isotherm parameters of P adsorption by OL-APTES-H+.

Isotherm model	Parameters	Temperature (K)		
		298	308	318
Langmuir	q_exp_ (mg/g)	18.61	18.13	17.84
	q_max_ (mg/g)	22.14	21.73	21.74
	K_L_ (L/mg)	0.038	0.035	0.032
	$R_{L}^{2}$	0.996	0.997	0.997
Freundlich	K_F_ (mg.(mg/L)^1/n^/g)	3.79	4.64	4.28
	n	3.17	3.75	3.58
	$R_{F}^{2}$	0.877	0.922	0.928

Thermodynamic parameters, including ΔG°, ΔH°, and ΔS°, were determined using Van't Hoff's equations to provide deeper insight into the kinetics of the adsorption process [49]. The resultant values are tabulated in Table 4. The determination of the Ka value followed the dimensionless transformation of K_L_ [50]. The graphical representation of ln Ka versus 1/T for phosphate uptake is depicted in Fig. S3. The calculated ΔH° value [−6.76 kJ/mol] suggests an exothermic nature of the adsorption process, favouring lower temperatures for effective P adsorption [51]. The observed decline in adsorption with increasing temperature during isotherm studies corroborates this observation. The negative ΔG° values, escalating with temperature, affirm the spontaneous nature of the process [52]. The positive ΔS° value also implies increased system disorder upon phosphate entrapment within the OL-APTES-H+ adsorbent, facilitating spontaneous adsorption. These findings collectively indicate that P adsorption on OL-APTES-H+ is both exothermic and spontaneous.

**Table 4:** Thermodynamic parameters for adsorption by OL-APTES-H+.

T (K)	K_a_	ΔG°(KJ/mol)	ΔH°(KJ/mol)	ΔS° (KJ/(mol ·k))
298	1.17 × 10^3^	−17.51	−6.76	0.03
308	1.18 × 10^3^	−17.88	–	–
318	0.98 × 10^3^	−18.23	–	–

A comparative analysis of the results obtained in this study with various biosorbents reported in the literature for phosphate removal in simulated wastewater reveals that OL-APTES-H+ exhibited higher phosphate adsorption capacity than modified biochar and activated carbon [Table 5]. This indicates that the simple strategy of functionalizing lignin can serve as an efficient approach for phosphate adsorption. While some adsorbents may demonstrate higher adsorption capacities than OL-APTES-H+, this study underscores the advantage of using a metal-free, single-step modification process, offering a sustainable and effective method for lignin functionalization with competitive adsorption performance.

**Table 5:** Comparative analysis of phosphate adsorption by OL-APTES-H+ and reported biosorbents.

Biosorbent used	Functionalization	Adsorption capacity (mg/g)	Refs
Industrial alkali lignin	Amination with diethylenetriamine in the presence of epichlorohydrin	46.28	[25]
Biogas residue	Amination Epichlorohydrin, N,N-dimethylformamide and Ethylenediamine	34.40	[53]
Bamboo biomass Biochar	Amination with polyethyleneimine	6.25	[54]
Rape straw biochar	Nanoscale zero-valent iron	12.56	[55]
Pinewood waste	Pyrolysis	20.5	[56]
Bamboo activated carbon	zirconia chloride octahydrate and Cetyltrimethylammonium bromide	4.74	[57]
Wheat straw	Amination with Epichlorohydrin Ethylenediamine and Diethylamine followed by Surface Imprinting with Phosphate Anions	10.32	[58]
Organosolv lignin	APTES-HCl	21.12	Present study

#### 3.2.3 Effect of coexisting ions

It is crucial to understand the effect of phosphate adsorption onto OL-APTES-H+ in the presence of co-existing ions, as real wastewater samples contain various ions that may hinder phosphate removal. Wastewater typically consists of a mixture of ions that could compete with phosphate for adsorption sites. Therefore, the interference of anions on phosphate adsorption by OL-APTES-H+ was examined using potassium chloride, potassium nitrate, and potassium sulfate as sources of Cl⁻, N $O_{3}^{-}$, and S $O_{4}^{2-}$, respectively, by adding 10 mg/L of each anion solution to the 50 mg/L phosphate solution. The presence of chloride and nitrate improved phosphate removal by 5.1 % and 2.2 %, respectively. This effect is likely attributed to the higher binding affinity of phosphate to OL-APTES-H+, which facilitates the displacement of Cl⁻ and NO₃⁻ ions from the adsorption sites, allowing phosphate to occupy the active sites on the surface. The adsorption of nitrate and chloride ions onto the quaternary ammonium groups can be effectively replaced by phosphate, forming a more stable structure [59]. In contrast, divalent sulfate ions reduced phosphate removal by 4.3 %, likely due to stronger competition for adsorption sites and greater ionic strength compared to monovalent ions [60]. Thus, phosphate adsorption by OL-APTES-H+ is enhanced in the presence of monovalent anions and attenuated by divalent anions [Fig. S4].

#### 3.2.4 Evaluation of modified lignin for phosphate removal from industrial dairy wastewater

The investigation focused on assessing the adsorption behavior of OL-APTES-H+ across diverse wastewater streams from the dairy processing industry, each with varying phosphate content. The wastewater samples [WW1 and WW2] used in the study were taken from milk protein production plants from the Irish dairy processing industry. WW1 had a phosphate concentration of 189–196 mg/L at pH 4.5, while WW2 had 186–200 mg/L at pH 2.6. The adsorbent OL-APTES-H+ exhibited a notable 26.3 % removal efficiency at a 0.5 g/L adsorbent loading during a contact time of approximately 1 h for WW1. In contrast, WW2 displayed a considerably lower removal efficiency of around 3.3 %. Subsequently, adhering to industry specifications, the adsorbent loading in both streams was increased to 100 g/L. Results indicated that the WW1 achieved a robust phosphorus removal efficiency of 58 % after mixing with adsorbent, while the WW2 demonstrated a 30 % phosphate removal efficiency. These findings underscore the exceptional efficacy of OL-APTES-H+ in actual wastewater samples at elevated dosages, suggesting a potential alternative to conventional metal-based adsorbents and flocculants. The large-scale application of biobased adsorbents offers a promising pathway for valorizing phosphate-adsorbed sludge into nutrient-rich fertilizer, aligning with established industry standards and supporting critical agricultural productivity needs. This strategy aligns with Ireland's Food Vision 2030 objectives for sustainability and resource efficiency, fostering a circular economy by converting waste by-products into valuable agricultural inputs. By improving soil health, decreasing reliance on synthetic fertilizers, and delivering positive environmental and community outcomes, this approach provides a robust framework for advancing sustainable agricultural practices [61].

#### 3.2.5 Desorption studies

Recyclability and re-usability are of paramount importance to ensure the adaptability of lignin-based adsorbents on a larger scale. The regeneration process should be cost-efficient, effective, and maintain the structural integrity of the adsorbent. In this context, the desorption rates of phosphates are a key factor in determining the adsorbent reusability. A desorption study was conducted at various time intervals [1 h, 3 h, and 4 h] using 0.1 mol/L NaOH solution. Results demonstrated a rapid increase in the desorption rate as time progressed, with the desorption efficiency increasing from 38.6 % after 1 h, to 89.9 % at 3 h, and reaching 98.9 % at 4 h [Fig. S5]. This suggests that OL-APTES-H+ has significant potential for reuse for phosphate recovery [62]. These findings support the notion that OL-APTES-H+ could be a viable adsorbent in large-scale operations, where desorption and recycling are critical to economical and sustainable operations.

## 4 Adsorption mechanism

The adsorption mechanism of OL-APTES-H+ for phosphate removal is a multi-step process involving electrostatic attraction, surface complexation, and chemisorption. Functionalization with APTES introduces amine groups [-NH₂] onto the lignin structure, which, upon protonation with HCl, convert into positively charged ammonium [-NH₃⁺] groups. This modification significantly enhances the surface charge of OL-APTES-H+, as confirmed by zeta potential measurements, which show that the material remains highly positive below pH 10, facilitating the attraction of negatively charged phosphate ions. In aqueous environments, phosphate exists in various forms, with H₂P $O_{4}^{-}$ being the dominant species at pH 5, where adsorption capacity is highest. The strong electrostatic attraction between H₂P $O_{4}^{-}$ ions and OL-APTES-H+ initially drive a rapid adsorption phase due to the high surface charge density of OL-APTES-H+, creating a steep potential energy gradient that accelerates surface-level adsorption onto active sites. As adsorption progresses, site saturation reduces the potential energy difference between the adsorbent and adsorbate, slowing the adsorption rate. At this stage, intraparticle diffusion becomes the dominant mechanism, as phosphate ions gradually migrate into the inner layers of OL-APTES-H+, a process that occurs more slowly than surface adsorption. Eventually, dynamic equilibrium is reached, where the rates of adsorption and desorption stabilize.

In this study, kinetic data were fitted to the pseudo-first-order and pseudo-second-order models, with the latter exhibiting a higher correlation coefficient [R^2^ close to 1]. This strong correlation clearly indicates that chemisorption is the dominant adsorption process. However, kinetic modelling should not be relied upon exclusively to confirm the mechanism [63]. To gain a comprehensive understanding of the adsorption mechanism, SEM imaging, EDS mapping, and various spectroscopic techniques [including FTIR and XPS] were employed. These methods provided compelling evidence of phosphate binding through electrostatic interactions and the formation of covalent bonds with functional groups on OL-APTES-H+.

**Fig. 8:**
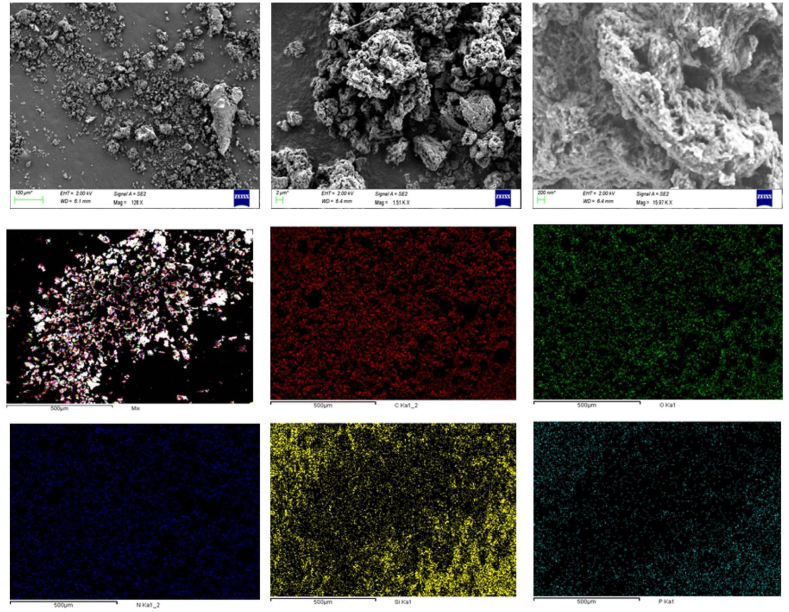
SEM images and EDS mapping of OL-APTES-H + -P.

**Fig. 9:**
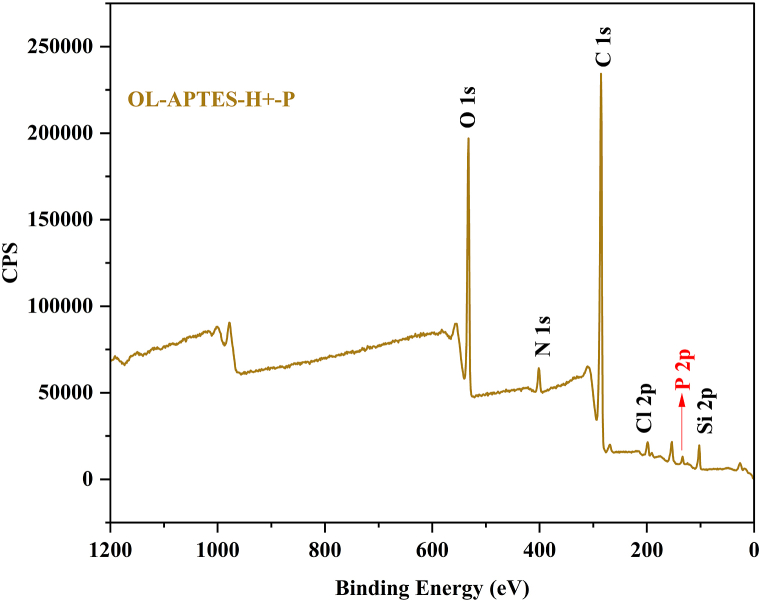
XPS spectra of OL-APTES-H + -P.

SEM imaging [Fig. 8] supports this conclusion by revealing morphological changes in OL-APTES-H + -P after adsorption, where the adsorbent appears more angular and blocky, indicating phosphate loading alters its structural characteristics [25]. Additionally, EDS mapping confirms phosphorus atoms on the surface, reinforcing the conclusion that adsorption occurs through strong interactions between phosphate and OL-APTES-H+. Spectroscopic analyses further confirm phosphate immobilization via chemisorption, as evidenced by changes in characteristic functional group vibrations and shifts in binding energies. FTIR spectra [Fig. S6**]** reveal the appearance of a new O-P-O asymmetric stretching peak at 1030 cm⁻^1^**,** confirming phosphate binding, along with Si-O-P peaks at 951 cm⁻^1^, suggesting covalent phosphate bonding to silanol groups [64]. Additionally, a decrease in the ammonium [-NH₃⁺] peaks at 3286 cm⁻^1^ and 3255 cm⁻^1^ after adsorption indicates that these functional groups actively participate in phosphate capture, likely via electrostatic interactions [59]. XPS analysis [Fig. 9, Fig. S1] provides further confirmation of chemisorption, with the Si 2p peaks at 102.7 eV [Si–O–C] and 105.3 eV [(P=O)–O–Si]**,** strongly indicating covalent bonding between phosphate and silanol groups on OL-APTES-H + [65]. Additionally, the N 1s peak at 401.3 eV**,** corresponding to protonated amines, confirms that ammonium groups play a key role in electrostatic adsorption [66]. The P 2p peak at 134.0 eV, with a phosphorus content of 0.85 %, provides direct evidence of successful phosphate incorporation onto OL-APTES-H+.

**Fig. 10:**
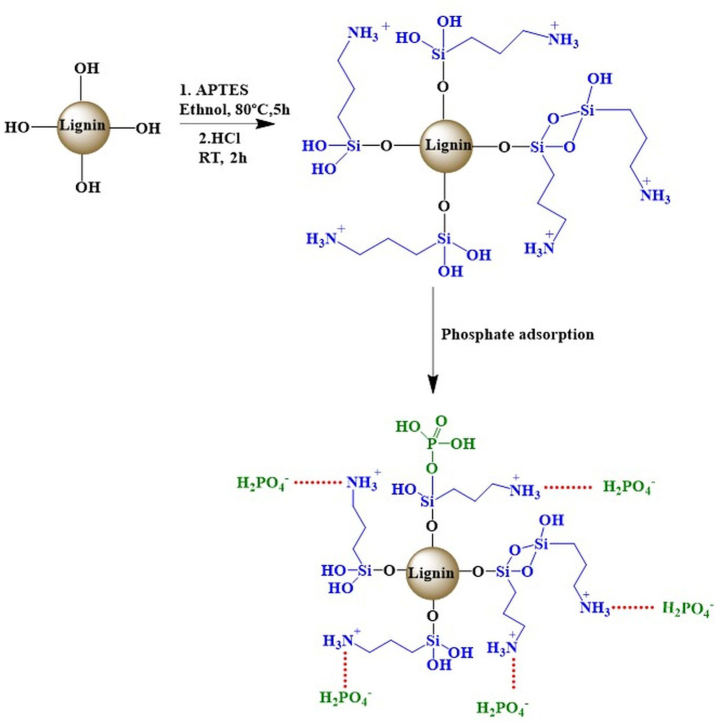
Proposed adsorption mechanism of OL-APTES-H+.

These combined findings from kinetic modeling, SEM, EDS, FTIR, and XPS analyses confirm that phosphate adsorption on OL-APTES-H+ is governed by a dual mechanism of electrostatic attraction and chemisorption. The electrostatic interaction between H₂P $O_{4}^{-}$ and protonated amine groups [-NH₃⁺] initiate the adsorption process, while chemisorption through covalent bond formation stabilizes phosphate immobilization [Fig. 10].

## 5 Conclusion

In this study, OL was successfully functionalized with APTES and protonated using dilute HCl treatment, resulting in an efficient adsorbent for phosphate removal from aqueous solutions. Characterization through SEM, EDS, FTIR, XPS, pH_pzc_, and zeta potential analysis confirmed the successful modification of OL. The maximum phosphate adsorption capacity of OL-APTES-H+ was observed at 298 K and pH 5. Kinetic studies demonstrated that the pseudo-second-order model best describes the adsorption process, indicating that both chemisorption and electrostatic attractions contribute to phosphate binding. Analytical techniques further validate this mechanism, confirming strong and stable phosphate immobilization on OL-APTES-H+. The intraparticle diffusion model indicated multiple adsorption stages, beginning with a rapid phase dominated by surface charge interactions, followed by a slower phase controlled by intraparticle diffusion, with equilibrium reached after 60 min. The adsorption isotherms were effectively modeled by the Langmuir equation, suggesting a monolayer adsorption mechanism and an exothermic, spontaneous process, as demonstrated by thermodynamic parameter analysis. The presence of coexisting ions demonstrated an enhancing effect from monovalent ions and a negative effect from divalent ions on phosphate removal efficiency. Furthermore, OL-APTES-H+ exhibits high phosphate desorption efficiency, indicating economical adsorption process for the recovery of phosphates and scalability. Moreover, experiments with real dairy wastewater confirmed its high phosphate removal efficiency, and the resulting sludge could be potential as a soil conditioner and slow-release fertilizer, underscoring its dual role in sustainable wastewater treatment and nutrient recovery.

## CRediT authorship contribution statement

**Minu Masliha:** Writing – original draft, Methodology, Investigation, Formal analysis, Conceptualization. **Mukesh Padnekar:** Writing – review & editing, Methodology, Conceptualization. **Jessica De Micco:** Writing – review & editing, Formal analysis. **Siva Ponnupandian:** Writing – review & editing, Formal analysis. **Kona Mondal:** Writing – review & editing, Formal analysis. **Ramesh Babu Padamati:** Writing – review & editing, Validation, Supervision, Funding acquisition, Conceptualization.

## Funding

This work was supported by the Department of Agriculture Food and the Marine, Ireland, NXTGENWOOD (grant code 2019PROG7040), 10.13039/100018175Dairy Processing Technology Centre (10.13039/100018175DPTC), funded by 10.13039/501100001588Enterprise Ireland (Grant Number TC2020 0028). KM would like to acknowledge the funding support from the European Union's Horizon research innovation program under grant agreement No. 101084437(IMPRESS).

## Declaration of competing interest

The authors declare the following financial interests/personal relationships which may be considered as potential competing interests:Ramesh Babu Padamati reports financial support was provided by Department of Agriculture Food and the Marine, Ireland. Ramesh Babu Padamati reports a relationship with 10.13039/100018175Dairy Processing Technology Centre (10.13039/100018175DPTC), funded by 10.13039/501100001588Enterprise Ireland that includes: employment. NA If there are other authors, they declare that they have no known competing financial interests or personal relationships that could have appeared to influence the work reported in this paper.
