# Supplementary material for: Functionalized organosolv lignin grafted with 3-aminopropyltriethoxysilane: A bio-based adsorbent for phosphate recovery from dairy wastewater
Source: Heliyon. 2025 Feb 8;11(4):e42559. doi: 10.1016/j.heliyon.2025.e42559 (PMC11870260; doi:10.1016/j.heliyon.2025.e42559)
Supplement: Multimedia component 1 [file mmc1.docx]

**Functionalized Organosolv Lignin Grafted with 3-Aminopropyltriethoxysilane: A Bio-based Adsorbent for Phosphate Recovery from Dairy Wastewater**

*Minu Masliha^1,2^, Mukesh Padnekar^2,3,4^ Jessica De Micco^1,2^, Siva Ponnupandian^1,2^, Kona Mondal^1,2^, Ramesh Babu Padamati^1,2,4 *^*

^1^ School of Chemistry, CRANN, Trinity College Dublin, D02 PN40 Dublin, Ireland

^2^ AMBER, SFI Research Centre for Advanced Materials and Bioengineering Research, D02 PN40 Dublin, Ireland SFI Research Centre, Trinity College Dublin, Dublin, Ireland

^3^ School of Physics, CRANN, Trinity College Dublin, D02 PN40 Dublin, Ireland

^4^ Dairy Processing Technology Centre (DPTC), University of Limerick, V94 T9PX Limerick, Ireland

**Supporting information**


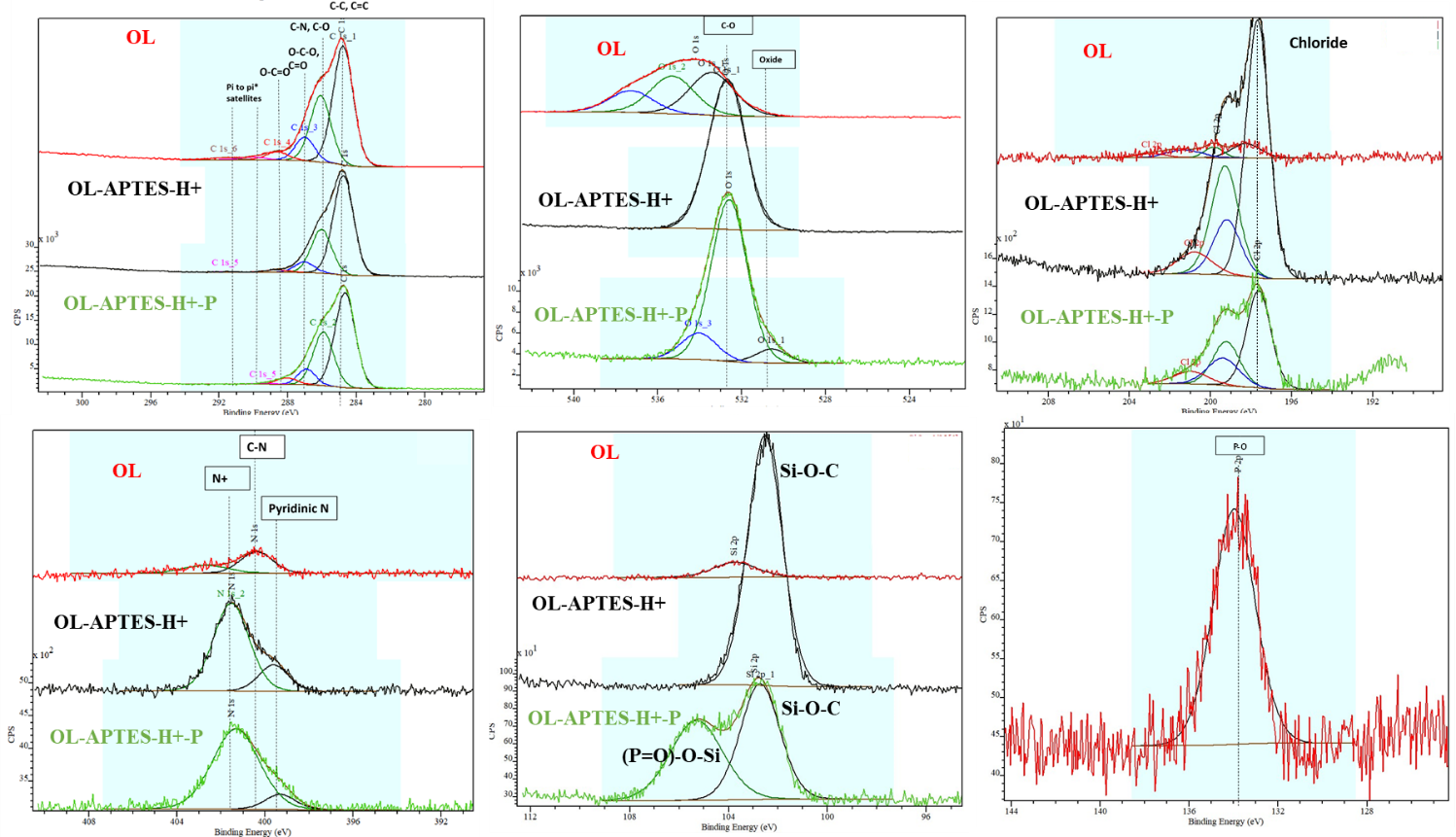


Figure S1 XPS spectra of OL, OL-APTES-H+ and OL-APTESH+-P

*
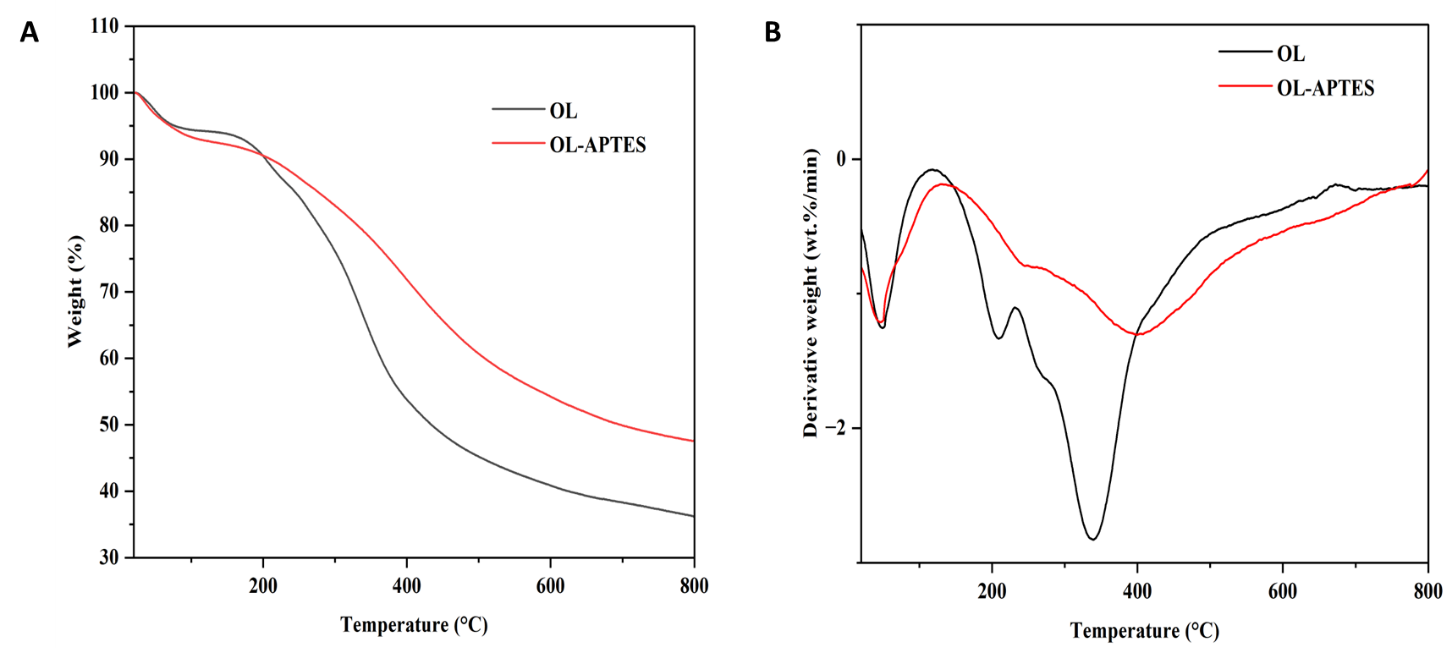
*

Figure S2 TG and DTG curves (A and B) of OL and OL-APTES

Table S 1 A comparison of kinetic data for phosphate adsorption from various adsorbents was reported in the literature.

| **Adsorbent** | **Best fit Model** | **Adsorbent dosage (g/L)** | **P concentration (mg/L)** | **R^2^** | **Reference** |
| --- | --- | --- | --- | --- | --- |
| Aminated lignin | Pseudo second order | 0.5 | 10 | 0.990 | 2 |
|  |  |  | 30 | 0.989 |  |
|  |  |  | 50 | 0.992 |  |
| Fe_2_O_3_ porous microspheres modified pumice | Pseudo second order | 1 | 10 | 0.999 | 3 |
| Quaternized straw supported by La (OH)_3_ nanoparticles | Pseudo second order | 0.8 | 20 | 0.999 | 4 |
|  |  |  | 30 | 0.999 |  |
|  |  |  | 40 | 0.999 |  |
| Hierarchical ZnO/ZnFe_2_O_4_ yolk-shell | Pseudo second order | 0.8 | 50 | 0.983 | 5 |
| SA/PVA - La-biochar matrix | Pseudo second order | 0.5 | 20 | 0.999 | 6 |
| OL-APTES-H+ | Pseudo second order | 0.5 | 30 | 0.987 | This work |
|  |  |  | 50 | 0.996 |  |


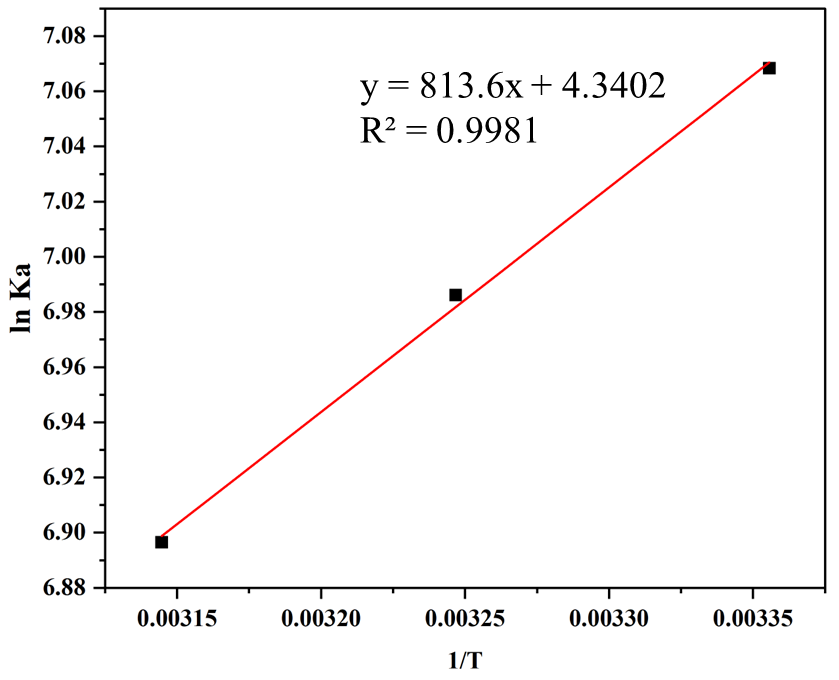


Figure S3 Graphical representation of ln Ka versus 1/T for phosphate uptake


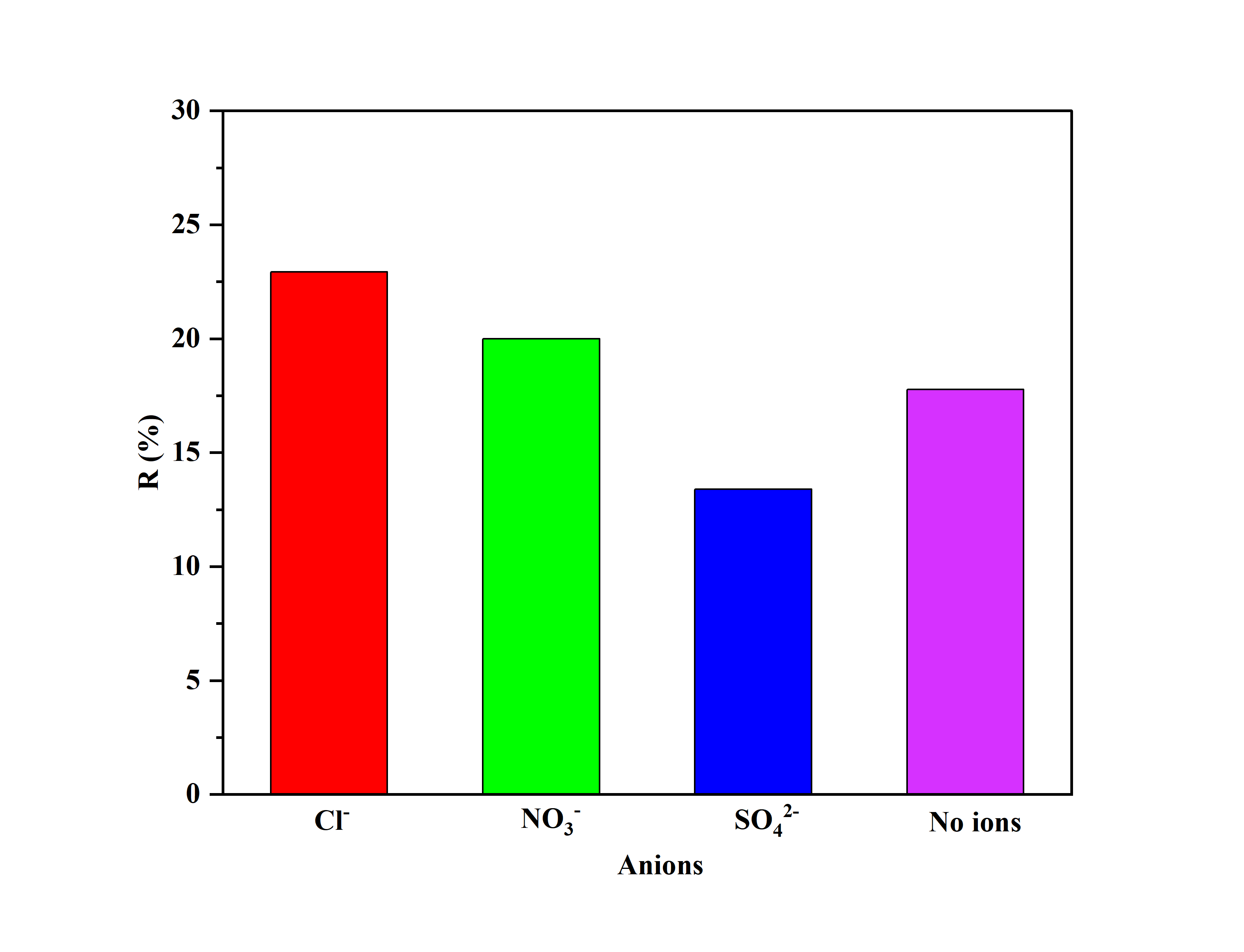


Figure S4 Effect of co-existing anions on the adsorption capacity of the OL-APTES-H+


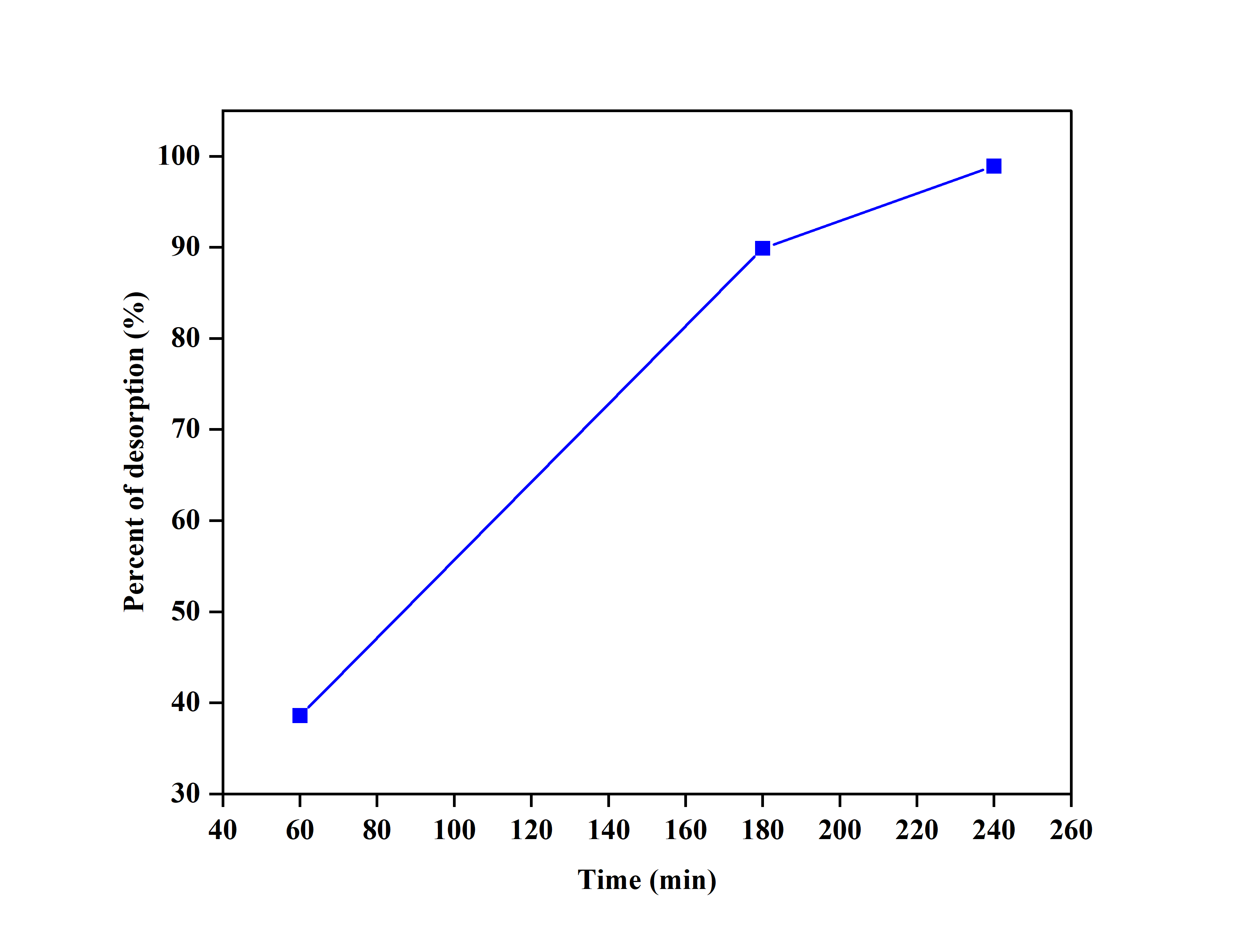


Figure S5 Desorption study of phosphates from OL-APTES-H+ at different time intervals


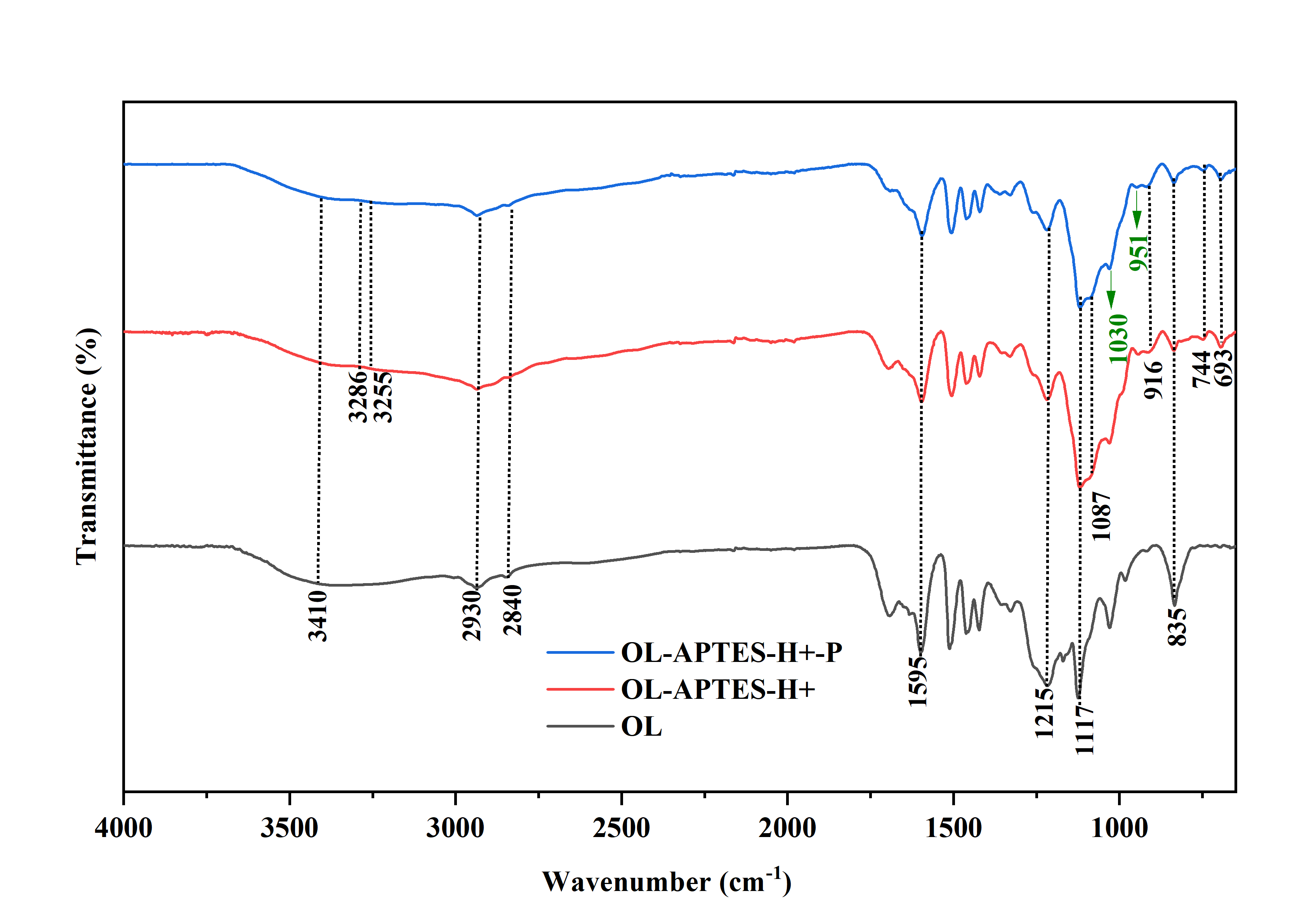


Figure S6 FTIR spectrum of OL, OL-APTES, OL-APTES-H+, and OL-APTES-H+-P

**References**

[1] S. Li, W. Xie, M. Wilt, J.A. Willoughby, O.J. Rojas, Thermally Stable and Tough Coatings and Films Using Vinyl Silylated Lignin, ACS Sustain. Chem. Eng. 6 (2018) 1988–1998. <https://doi.org/10.1021/acssuschemeng.7b03387>.

[2] Z. Wang, A. Abbas, H. Sun, H. Jin, T. Jia, J. Liu, D. She, Amination-modified lignin recovery of aqueous phosphate for use as binary slow-release fertilizer, Int. J. Biol. Macromol. 242 (2023) 124862–124862. <https://doi.org/10.1016/j.ijbiomac.2023.124862>.

[3] Y. Zhang, Y.-F. Xiao, G.-S. Xu, D.-C. Wang, J. Li, J. Huang, Z. Jin, Preparation of Fe2O3 porous microspheres modified pumice and its adsorption performance on phosphate removal, J. Environ. Chem. Eng. 11 (2023) 109995–109995. <https://doi.org/10.1016/j.jece.2023.109995>.

[4] W. Song, L. Zhang, B. Guo, Q. Sun, Z. Yu, X. Xu, Y. Zhao, L. Yan, Quaternized straw supported by La(OH)3 nanoparticles for highly-selective removal of phosphate in presence of coexisting anions: Synergistic effect and mechanism, Sep. Purif. Technol. 324 (2023) 124500–124500. <https://doi.org/10.1016/j.seppur.2023.124500>.

[5] P. Madhusudan, C. Lee, J.-O. Kim, Hierarchical ZnO/ZnFe2O4 yolk-shell adsorbent as a promising material for phosphate recovery and adsorption of organic pollutants from the simulated wastewater, Sep. Purif. Technol. 348 (2024) 127598–127598. <https://doi.org/10.1016/j.seppur.2024.127598>.

[6] B. Wang, X. Hu, L. Li, Y. Xie, R. Chen, W. Guo, H. Wang, M. Wang, J. Shi, L. Chen, D. Zhou, Enhanced phosphate removal by filler encapsulation and surface engineering using SA/PVA matrix: Fabrication optimization, adsorption behaviors and inner removal mechanism, J. Chem. Eng. 472 (2023) 145073–145073. https://doi.org/10.1016/j.cej.2023.145073.

[7] E.C. Lima, A. Hosseini-Bandegharaei, J.C. Moreno-Piraján, I. Anastopoulos, A critical review of the estimation of the thermodynamic parameters on adsorption equilibria. Wrong use of equilibrium constant in the Van’t Hoof equation for calculation of thermodynamic parameters of adsorption, J. Mol. Liq. 273 (2019) 425–434. <https://doi.org/10.1016/j.molliq.2018.10.048>.
